# Biodistribution of etoposide via intratumoral chemotherapy with etoposide-loaded implants

**DOI:** 10.1080/10717544.2020.1787558

**Published:** 2020-07-02

**Authors:** Chunsheng Wu, Xiangting Yi, Renzhi Xu, Maokuan Zhang, Yan Xu, Yan Ma, Li Gao, Zhengbao Zha

**Affiliations:** a School of Food and Biological Engineering, Hefei University of Technology, Hefei, PR China; b Laboratory of Pharmaceutical Research, Anhui Zhongren Science and Technology Co., Ltd, Hefei, PR China

**Keywords:** Etoposide, PLLA, implants, intratumoral chemotherapy, biodistribution

## Abstract

Etoposide (VP16) is the traditional antitumor agent which has been widely used in a variety of cancers. However, intravenous administration of VP16 was limited in clinical application because of its low aqueous solubility, poor bioavailability and dose-limiting adverse effects. Local chemotherapy with VP16-loaded drug delivery systems could provide a continuous release of drug at the target site, while minimizing the systemic toxicity. In this study, we prepared the poly-l-lactic acid (PLLA) based VP16-loaded implants (VP16 implants) by the direct compression method. The VP16 implants were characterized with regards to drug content, micromorphology, drug release profiles, differential scanning calorimetry (DSC) and Fourier transform infrared spectroscopy (FTIR) analyses. Furthermore, the biodistribution of VP16 via intratumoral chemotherapy with VP16 implants was investigated using the murine Lewis lung carcinoma model. Our results showed that VP16 dispersed homogenously in the polymeric matrix. Both *in vitro* and in *vivo* drug release profiles of the implants were characterized by high initial burst release followed by sustained release of VP16. The VP16 implants showed good compatibility between VP16 and the excipients. Intratumoral chemotherapy with VP16 implants resulted in significantly higher concentration and longer duration of VP16 in tumor tissues compared with single intraperitoneal injection of VP16 solution. Moreover, we found the low level of VP16 in plasma and normal organ tissues. These results suggested that intratumoral chemotherapy with VP16 implants enabled high drug concentration at the target site and has the potential to be used as a novel method to treat cancer.

## 1. Introduction

Systemic chemotherapy is the most common method used to treat cancer. However, many solid tumors show an increased interstitial fluid pressure (IFP) which forms the barrier for antitumor agents to reach tumor via transcapillary transport (Heldin et al., 2004). Moreover, systemic chemotherapy is often ineffective to solid tumors, mainly due to unfavorable pharmacokinetics, poor delivery, low local concentration and limited accumulation of antitumor drugs in target cells (Krukiewicz & Zak, 2016). High systemic doses of drug usually result in undesirable adverse effects to normal tissues (Saltzman & Fung, 1997). It is well known that traditional systemic chemotherapy employs high level of intravenous cytotoxic drugs and frequently causes severe systemic toxicity which limits the effectiveness and compromises the quality of life for patients (Goldberg et al., 2002; Garrastazu Pereira et al., 2016).

Local chemotherapy with drug delivery systems can provide continuous drug release and enables high drug concentration at the target site with minimal systemic toxicity compared with traditional chemotherapy. Furthermore, it is beneficial to prevent local recurrence of cancer after resection (Krukiewicz & Zak, 2016; Gao et al., 2019). The tumor core consists of chemotherapy resistant tumor cells, and intratumoral chemotherapy was considered as a method to sensitize the tumor to radiotherapy and systemic chemotherapy (Hohenforst-Schmidt et al., 2013).

Anticancer drug-loaded implant is considered to act as adjunctive therapy or an efficient alternative when the conventional therapies fail or can not be applied (Al-Zu'bi & Mohan, 2019). Implants for controlled drug delivery can improve the therapeutic efficacy of a medical treatment and reduce the toxicity simultaneously (Blanchemain et al., 2017). Gliadel^®^ (carmustine-loaded polymer wafer) is the first antitumor implant approved by US Food and Drug Administration for treatment of newly-diagnosed or recurrent malignant glioma (Gao et al., 2017). Clinical trials have shown that Gliadel^®^ implantation combined with radiotherapy and temozolomide significantly increased the overall survival (Ashby et al., 2016; Kumabe et al., 2018).

Etoposide (VP16) is the traditional anticancer drug that has been widely used to treat a variety of cancers in children and adults. VP16 was derived from podophyllotoxin and first synthesized in 1966. It was the first agent recognized as a topoisomerase II (Topo II) inhibiting anticancer drug (Hande, 1998; Montecucco et al., 2015). Mammals have two Topo II isoenzymes: Topo IIα and Topo IIβ. VP16 targeting of Topo IIβ in differentiated tissues may account for much of the off-target toxicity of the drug (Montecucco et al., 2015).

Two commercial products of VP16 are available on the market nowadays: VP16 injections and the oral soft capsule. However, the low aqueous solubility of VP16 injections and the poor bioavailability and wide between-patient variability of oral VP16 capsules limit their clinical applications (Dong et al., 2013; Solano et al., 2013). Many VP16-loaded drug delivery systems have been reported to overcome the limitations of VP16 injections and capsules, including microemulsions, micelles, nanoparticles and liposomes (Najar & Johri, 2014). Furthermore, the co-delivery systems of VP16 have shown synergistic effects in cancer treatment (Yuan et al., 2017; Kumar et al., 2018; Zhang et al., 2018; Li et al., 2019; Popova et al., 2019).

In this study, the poly-l-lactic acid (PLLA) based VP16-loaded implants (VP16 implants) were prepared and characterized regarding drug content, micromorphology, drug release profiles, differential scanning calorimetry (DSC) and Fourier transform infrared spectroscopy (FTIR) analyses. To gain further information of the biodistribution of VP16 after intratumoral chemotherapy with the VP16 implants, we established the Lewis lung carcinoma model in mice and detected VP16 concentration in plasma and tissues of the mice using the ultra-performance liquid chromatography-tandem mass spectrometry (UPLC-MS/MS) method.

## 2. Materials and methods

### 2.1. Chemicals and animals

Etoposide (Lot:100388-200401, purity ≥ 98%) was purchased from China’s Food and Drug Inspection Institute. Teniposide (Lot: E1528013, purity ≥ 98%) was used as internal standard (IS) and purchased from Aladdin Reagent Co., Ltd. (Shanghai, China). Poly-l-lactic acid (PLLA) (Molecular Weight, Mw = 17087) was generously provided by Anhui Zhongren Science and Technology Co., Ltd. (Anhui, China). Polyethylene glycol 4000 (PEG4000) was purchased from Beijing Huiyou Chemical Co., Ltd. (Beijing, China). Etoposide injection was purchased from Jiangsu Hengrui Medicine Co., Ltd. (Jiangsu, China). HPLC grade formic acid was obtained from Tianjin Kermel Chemical Reagent Co., Ltd. HPLC grade methanol, acetonitrile and trichloromethane were obtained from Tedia Company, Inc. The high-glucose Dulbelcco’s Modified Eagle’s Medium and fetal calf serum was purchased from Gibco Ltd. (New York, NY, USA). Ultra-pure water was obtained in milli-Q system from Millipore (Bedford, MA). All other chemicals were of analytical grade.

The murine Lewis lung carcinoma cells were from the Cell Bank of the Chinese Academy of Sciences (Shanghai, China). The healthy Kunming mice and the Wistar rats were purchased from Experimental Animal Center of Anhui Medical University (Anhui, China). The animals were kept at constant temperature (23 °C ± 2 °C) and humidity (50 ± 5%) and had free access to clean food and water. The animal experiments were complied with the ARRIVE guidelines and carried out in accordance with the U.K. Animals (Scientific Procedures) Act, 1986 and associated guidelines.

### 2.2. Cell culture and establishment of Lewis lung carcinoma (LLC) model in mice

The murine Lewis lung carcinoma cells were cultured in high-glucose Dulbelcco’s Modified Eagle’s Medium supplemented with 10% fetal calf serum. The cells were placed in a 75 cm^2^ cell-culture flask at 37 °C with 5% carbon dioxide. The cell suspension was adjusted to 1 × 10^7^ cells/mL and 100 µl of the suspension was injected subcutaneously into the right posterior flank of the mice to establish the LLC model.

### 2.3. Preparation and characterization of VP16 implants

#### 2.3.1. Preparation of the VP16 implants

The preparation process of the VP16 implants was modified on our previous method (Gao et al., 2017). Briefly, the dry powders of etoposide, PLLA and PEG4000 were sieved separately. Then the mixture containing 40% etoposide, 50% PLLA and 10% PEG4000 (w/w) were thoroughly blended and further molded into cylindrical implants using the drug press machine (ZR-YYJB, Anhui, China) under the pressure of 20–25 MPa.

#### 2.3.2. Determination of drug content of the VP16 implants

The determination of drug content of the VP16 implants was performed according to the instructions described in the Chinese Pharmacopeia (The Pharmacopoeia Commission of the People’s Republic of China, 2019). Ten implants were selected and weighed. Then each implant sample was grounded and dissolved in the mixture of acetic acid (pH 4.0) and acetonitrile (70:30, v/v) in an ultrasonic water bath for 20 min. The suspension was transferred to a sterile centrifuge tube and centrifuged at a speed of 12000 rpm for 10 min. Subsequently, an aliquot of the supernatant (20 µl) was analyzed by the high performance liquid chromatography (HPLC) method.

#### 2.3.3. Micromorphology of the VP16 implants

Scanning electron microscopy (SEM) was used to observe the micromorphology of the VP16 implants. The JEOL JSM-6490LV scanning electron microscope (JEOL, Tokyo, Japan) was operated at an acceleration voltage of 20 kV. Prior to imaging, the VP16 implants were placed on metal sample holders and coated with gold for 90 s at 20 mA using the JEOL JFC-1600 auto fine coater (JEOL, Tokyo, Japan). The external and internal morphologies were imaged at ×1500 magnification.

#### 2.3.4. Drug release profiles of the VP16 implants

We examined the drug release both *in vitro* and *in vivo* in the study. The *in vitro* drug release was performed by the rotating basket method. One VP16 implant sample was placed in 10 mL release medium consisting of phosphate-buffered saline (PBS pH 5.0) and isopropyl alcohol (93:7, v/v). The rotating speed of the basket was set at 130 rpm and the temperature was maintained at 37 °C ± 0.5 °C. At different time intervals (2, 4, 8, 24, 48, 72, 96, 120, 144, 168 and 192 h), 3 mL of the sample was withdrawn, centrifuged at 12000 rpm for 10 min and stored at 4 °C until analysis. Then 3 mL of fresh release medium was immediately added back to the dissolution flask to maintain the constant sink condition. The test was performed in sextuple for each batch.

To determine the *in vivo* drug release of the VP16 implants, one VP16 implant was inserted intramuscularly into the right leg of the Wistar rats. At 1, 5, 10, 15, 20, 25, 30, 40 and 45 days after implantation, the rats were euthanized by CO_2_ asphyxiation, then the VP16 implant was retrieved, dried and stored at 4 °C until analysis. Three rats were used at each time point. The amount of drug in the residue implant was analyzed by HPLC method. The *in vivo* drug release of the VP16 implant was calculated according to the formula as follows:

$$\text{Etoposide release percentage}\left(\%\right)=\frac{\text{initial etoposide amount}-\text{residual etoposide amount}}{\text{initial etoposide amount}}\times 100\%$$

#### 2.3.5. Differential scanning calorimetry (DSC) analysis

DSC analysis was carried out with a thermal analysis instrument (Q2000; TA Instruments, New Castle, DE, USA). Samples (about 5 mg) of the VP16 implants, pure VP16, pure PLLA, pure PEG4000, and physical mixture of VP16, PLLA and PEG4000 (4:5:1, w/w) were sealed in aluminum pans and measured by DSC at a heating rate of 10 °C min^−1^ over a temperature range of 25 °C–500 °C. High purity nitrogen was used as the purge gas at a flow rate of 50 mL/min.

#### 2.3.6. *Fourier transform infrared spectroscopy* (*FTIR) analysis*

Samples of the VP16 implants, pure VP16, pure PLLA, pure PEG4000, and physical mixture of VP16, PLLA and PEG4000 (4:5:1, w/w) were analyzed by FTIR. The infrared spectra were generated in the FTIR spectrophotometer (Nicolet 6700; Thermo Fisher Scientific, Waltham, MA, USA). Measurements were carried out using the attenuated total reflectance technique. Each spectrum was a result of 32 scans with a resolution of 4 cm^−1^.

#### 2.3.7. *In vivo* degradation of PLLA

PLLA was fabricated as solid cylinder using the method for preparation of VP16 implants. One PLLA sample was implanted subcutaneously on the back of the Wistar rat. The rats were sacrificed at different time points (2, 4, 7, 10, 14, 18, 22, 26, 31 and 41 weeks) post implantation. Then the PLLA sample was retrieved, cleaned and dried under vacuum. Then the weight loss of PLLA was detected and expressed as the ratio between weight loss and the initial weight of the PLLA (*n* = 3).

### 2.4. The HPLC method for determination of VP16 in the implants

The drug content in the VP16 implants was detected by HPLC method. The HPLC system was equipped with two LC-15C pumps, a SPD-15C essential UV detector and a CTO-15C essential column oven (Shimadzu, Japan). The analytical column (250 mm × 4.6 mm, 5 µm particle size) was maintained at 25 °C in the column oven (Hypersil BDS C6H5 column). The mobile phase consisted of 0.2% acetic acid (pH 4.0) and acetonitrile (70:30, v/v) and the flow rate was 1.5 mL/min. The injection volume was 20 µl and the UV detection was performed at 254 nm.

### 2.5. Biodistribution study in Lewis lung carcinoma-bearing mice

Eighty healthy Kunming mice weighing 18–22 g were used in the experiment to establish the LLC model. When the tumor volume reached 300–400 mm^3^, the Lewis lung carcinoma-bearing mice were randomly divided into two groups (*n* = 40 per group): (i) the mice received intraperitoneal injection of VP16 solution at the dose of 75 mg/kg. (ii) the mice were implanted with the VP16 implants intratumorally at the dose of 75 mg/kg. During the experiment, the mice were sacrificed and the blood samples were collected from the eye socket vein into heparinized centrifuge tube at the indicated time intervals (2, 6, 12, 24, 72, 120, 168 h after treatment). After centrifuging at 3000 rpm for 10 min, the supernatant was transferred to a clean centrifuge tube and stored at −80 °C. Moreover, the tissues of tumor, heart, liver, spleen, lung and kidney were isolated at the predetermined time points (1, 3, 5, 7, 10 days after treatment), rinsed with ultra-pure water, dried and stored at −80 °C until analysis. Five animals were used at each time point.

### 2.6. The UPLC-MS/MS assay for quantification of VP16 in plasma and tissues

We established an UPLC-MS/MS method to quantitate VP16 in plasma and tissues of Lewis lung carcinoma-bearing mice. The established UPLC-MS/MS method was fully validated in compliance with FDA guidelines for bioanalytical method validation (U.S. Department of Health and Human Services, 2018).

#### 2.6.1. UPLC-MS/MS instrumentations

Chromatographic separation was performed using the Waters ACQUITY ultra-performance liquid chromatography (Waters Corp., MA, USA) and the mass analysis was carried out with the Quattro-Premiere system (Waters Corp., MA, USA). Data were acquired and processed by the MassLynx software (version 4.1). The column used was Acquity UPLC BEH C18 (Waters Corp., 50 mm × 2.1 mm, 1.7 µm particles).

#### 2.6.2. LC And MS conditions

Ultra-pure water (A) and acetonitrile (B), both containing 0.1% formic acid, were used for separating the analytes on the BEH C18 column at a flow rate of 0.2 mL/min under gradient elution: 0 min, 30% B; 1 min, 70% B; 2.2 min, 70% B; 3.0 min, 30% B; 4.0 min, 30% B. The total run time was 4 min and the injection volume was 5 µl for each sample. The column oven temperature was set at 37 °C and the autosampler temperature at 10 °C.

The optimal conditions for analysis were as follows: capillary voltage 3.2 kV, cone voltage 20 V, desolvation gas (nitrogen) 350 °C and 700.0 L/hour, entrance potential 0 V, cell exit potential 1 V and collision energy 15 eV. Detection was performed using positive electrospray ionization (ESI) source via multiple reaction monitoring (MRM) mode. The MRM transitions were m/z 589.2 → 228.9 for etoposide and m/z 656.9 → 382.9 for IS.

#### 2.6.3. Preparation of plasma and tissue samples for UPLC-MS/MS analyses

An aliquot of 200 µl plasma was placed in a 2.0 mL centrifuge tube and mixed with 20 µl IS working solution (8 µg/mL). The mixture was extracted with 1 mL of trichloromethane by vortex-mixing at a high speed for 10 min, then the samples were centrifuged at 8000 rpm for 10 min. The phases were separated and the organic extracts were transferred to a new centrifuge tube, evaporated to dryness under nitrogen and reconstituted in 800 µl of acetonitrile-water (50:50, v/v). After vortex-mixing for 1 min, the supernatant was centrifuged at 13000 rpm for 10 min and 5 µl of it was injected for analysis.

To pretreat the tissue samples, the defined amount of tissues (tumor, heart, liver, spleen, lung and kidney) were accurately weighed (0.1 g) and homogenized in acetonitrile–water (50:50, v/v). After being centrifuged at 8000 rpm for 10 min, an aliquot of 200 µl tissue homogenate was mixed with 20 µl IS working solution (8 µg/mL) by vortex-mixing for 1 min, followed by addition of 1 mL trichloromethane and again vortexed for 10 min, centrifuged at 8000 rpm for 10 min. The organic extracts were transferred and evaporated to dryness under nitrogen. Then the residue was reconstituted in 800 μl acetonitrile–water (50:50, v/v). After a 1-min vortex mixing and a 10-min centrifugation at 13000 rpm, 5 µl of supernatant was injected for analysis.

### 2.7. Statistical analysis

All descriptive parameters were expressed as mean ± standard deviation and analyzed using the GraphPad Prism version 7.0 (GraphPad Software, Inc., La Jolla, CA, USA). The Student’s *t*-test was applied to investigate the differences between the two groups. The *p* value < .05 was considered as statistical significance.

## 3. Results

### 3.1. Preparation of the VP16 implants

The VP16 implants were prepared as cylinder with the mean length of 1.90 ± 0.09 mm and diameter of 0.90 mm (Supplementary Figure S1). In addition, the VP16 implants had an average weight of 1.52 ± 0.03 mg (*n* = 10).

### 3.2. Drug content of the VP16 implants

Ten VP16 implants were randomly selected and tested the content of VP16 by HPLC method stated in the Chinese Pharmacopeia. The mean value of drug content of the tested implants was (36.40 ± 0.07)%.

### 3.3. Micromorphology of the VP16 implants

**Figure 1.**
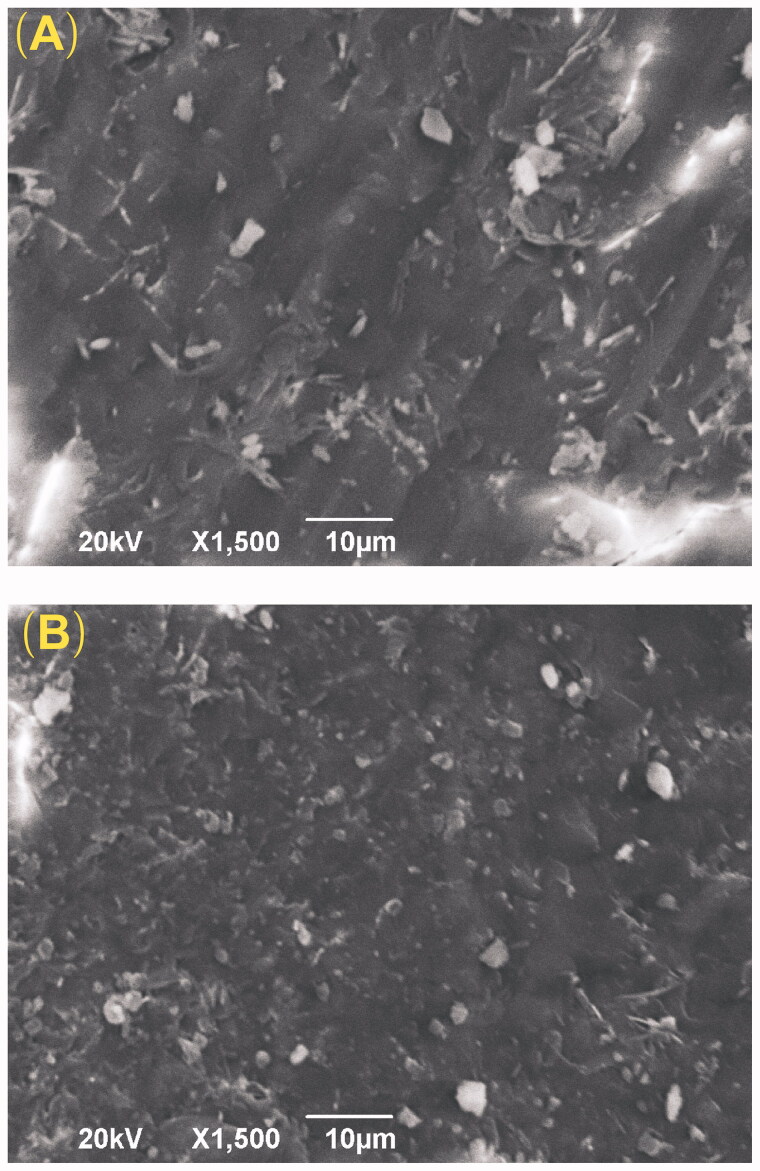
The SEM images of the VP16 implants (magnification ×1500). (A) The external surface of the VP16 implants. (B) The cross-section of the VP16 implants.

SEM was used to observe the microstructure of the VP16 implants. As shown in Figure 1, the external surface of the VP16 implants was smooth and homogenous. Additionally, the cross-section of the implants exhibited the homogenous appearance. We did not find obvious pores or channels in the SEM micrographs of the VP16 implants.

### 3.4. The *in vitro* and *in vivo* drug release profiles of the VP16 implants

**Figure 2.**
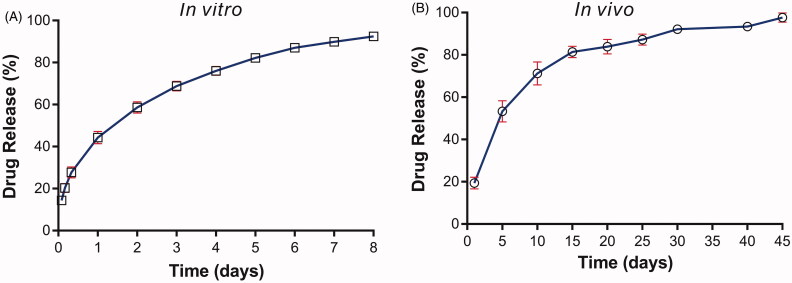
The drug release profiles of the VP16 implants. (A) The *in vitro* drug release profile of the VP16 implants (*n* = 6 for each time). (B) The *in vivo* drug release profile of the VP16 implants (*n* = 3 for each time). Data were shown as mean ± standard deviation.

The *in vitro* drug release studies were carried out using the dissolution apparatus. The solution consisting of PBS (pH5.0) and isopropyl alcohol (93:7, v/v) was used as the release medium. The result was illustrated in Figure 2(A). The implants released approximately 14% of VP16 in the first 2 h. The mean cumulative release percentage was 44% within 1 day. Then we observed the release rate steadily decreased and almost 80% of drug released from the VP16 implants in 4 days. Finally, the cumulative drug release approximately reached an average of 92% over 8 days.

To determine the *in vivo* drug release of the VP16 implants, one VP16 implant were inserted intramuscularly in each Wistar rat. Then the implant was collected on day 1, 5, 10, 15, 20, 25, 30, 40 and 45 post implantation. Furthermore, the drug content in the collected implant was detected and the drug release was calculated. As shown in Figure 2(B), 19.4% of VP16 was released from the implant on day 1. The implant released 53% of drug on day 5 and 71% of drug within 10 days. Then the drug release rate slowed down and the implant released VP16 continuously almost at a constant rate. Finally, we observed the complete drug release from the implant within 45 days.

### 3.5. DSC analysis of the VP16 implants

**Figure 3.**
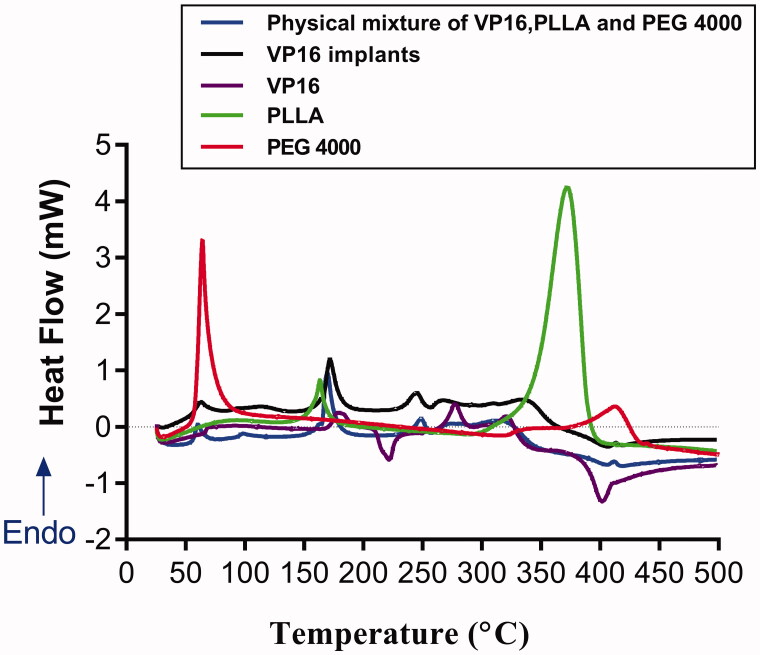
The DSC curves of the pure VP16, pure PLLA, pure PEG4000, VP16 implants and physical mixture of VP16, PLLA and PEG4000.

The thermal behaviors of pure PEG4000, pure PLLA, pure VP16, VP16 implants and physical mixture of VP16, PLLA and PEG4000 were analyzed by DSC (Figure 3). The DSC curve of pure PEG4000 exhibited a melting sharp endothermic peak centered at 60 °C. In the DSC curve of pure PLLA, we observed the shark endothermic peaks centered at 164 °C and 372 °C, respectively. The DSC curve of pure VP16 showed an obvious endothermic peak in the temperature between 169 °C and 192 °C, centered at about 180 °C, which was related to the melting of the drug. Then we observed an exothermic peak in the range of 211 °C to 230 °C. Furthermore, the second endothermic peak centered at about 276 °C was detected in the DSC curve of VP16 which may be attributed to the melting point of the newly formed etoposide (Solano et al., 2013). From the DSC curves of VP16 implants and the physical mixture of VP16 and excipients, we observed the similar thermal behaviors. Additionally, we did not find new endothermic or exothermic peaks in DSC curve of the VP16 implants.

### 3.6. FTIR analysis of the VP16 implants

**Figure 4.**
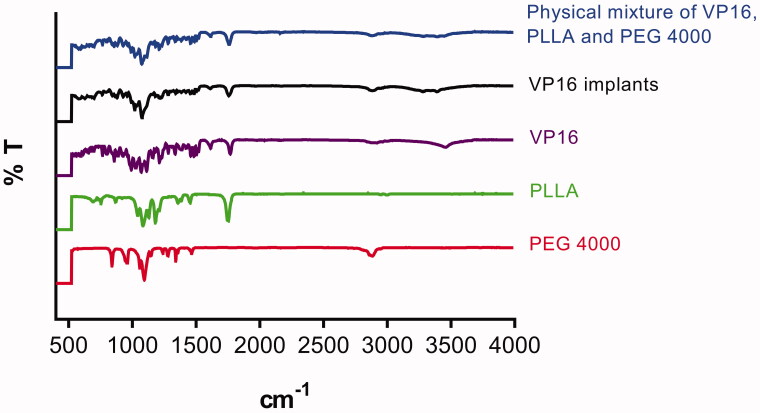
The FTIR spectra of the pure VP16, pure PLLA, pure PEG4000, VP16 implants and physical mixture of VP16, PLLA and PEG4000.

The FTIR study was used to describe the characteristic absorption bands at different frequencies for pure VP16, pure PLLA, pure PEG4000, VP16 implants and physical mixture of VP16, PLLA and excipients. The result was shown in Figure 4. From the FTIR spectrum of pure VP16, we observed the characteristic bands at 3455 cm^−1^, 1766 cm^−1^ and 1612 cm^−1^, respectively. Typical infrared absorption bands examined in the FTIR spectra of PLLA and PEG4000 were detected in the spectra of VP16 implants and the physical mixture of VP16 and excipients. Additionally, we did not find new absorption bands in the FTIR spectrum of VP16 implants.

### 3.7. The *in vivo* degradation of PLLA

The *in vivo* degradation of PLLA was analyzed by measuring the weight loss of the PLLA samples over 41 weeks. As shown in Supplementary Figure S2, Approximately 5% weight loss of the samples was observed within 4 weeks. Then the weight loss of PLLA increased very slowly. We found that the samples lost almost 45% of their original weight within 41 weeks.

### 3.8. Biodistribution of VP16 via intratumoral chemotherapy with the VP16 implants

**Figure 5.**
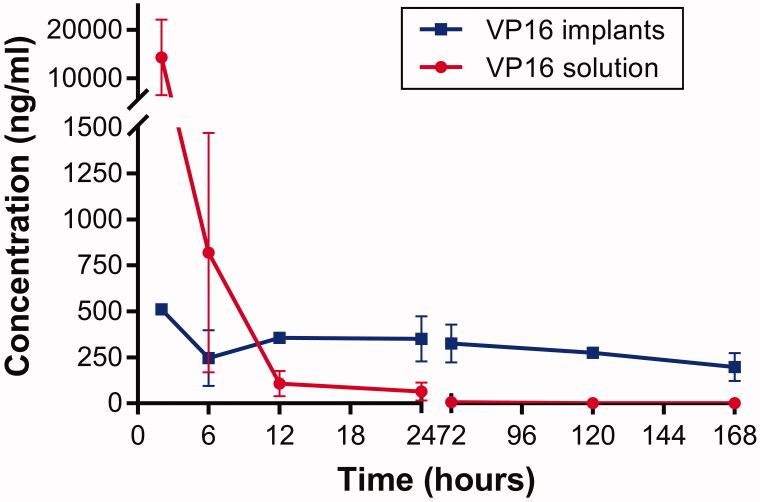
Plasma concentration–time curve for VP16 implants and VP16 solution.

The Lewis lung carcinoma-bearing mice were administered intratumoral chemotherapy with VP16 implants at the dose of 75 mg/kg. The mice in control group were intraperitoneally injected equivalent dosage of VP16 solution. The mice were sacrificed, and the blood was collected and centrifuged immediately at different time intervals after the treatment. The VP16 levels in plasma were measured and the result was presented in Figure 5. We detected high plasma level of VP16 within 2 h following intraperitoneal injection, then the concentration of VP16 declined sharply and below detection limit 24 h after treatment. The detected plasma concentration of VP16 was 510 ng/mL 2 h after intratumoral chemotherapy with VP16 implants. Moreover, the drug concentration in plasma declined slightly and maintained at a low level for a prolonged period of time.

**Figure 6.**
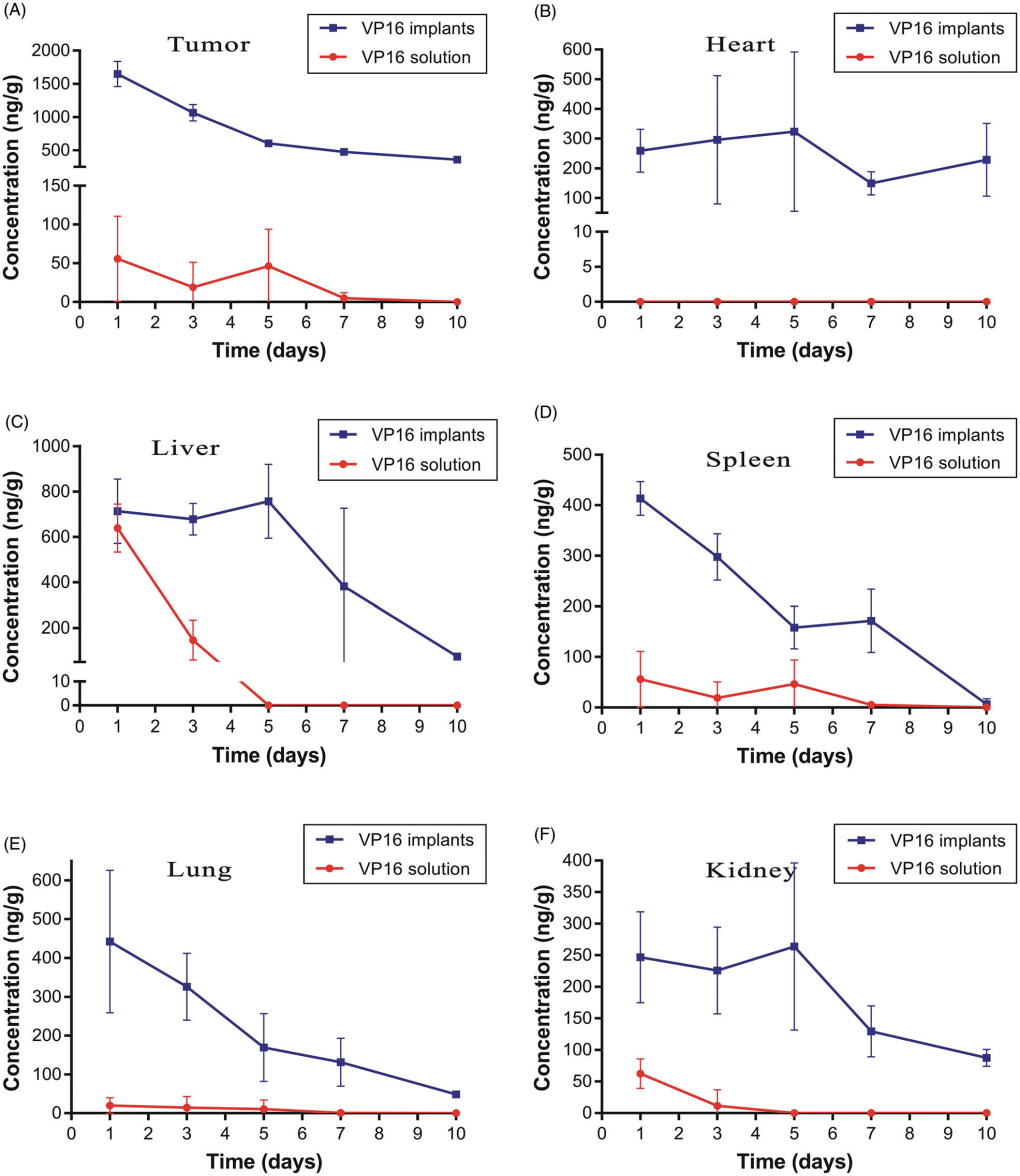
Tissue concentration–time curve for VP16 implants and VP16 solution. (A) Drug concentration in tumor at different time intervals. (B) Drug concentration in heart tissue at different time intervals. (C) Drug concentration in liver tissue at different time intervals. (D) Drug concentration in spleen tissue at different time intervals. (E) Drug concentration in lung tissue at different time intervals. (F) Drug concentration in kidney tissue at different time intervals.

To determine the VP16 concentration in tumor tissues, the mice were sacrificed at predetermined time intervals and the tumors were isolated. After removing the drug residues in the implantation site, VP16 concentration in tumor was detected using the UPLC-MS/MS method. As shown in Figure 6(A), the average concentration of VP16 in the tumor was 1647 ng/g on the first day after intratumoral chemotherapy with the VP16 implants. The drug level in tumor decreased slowly with the extension of time and the concentration of VP16 was still detectable even 10 days after the treatment. However, we detected the average concentration of VP16 below 55 ng/g in tumors of mice receiving intraperitoneal injection of VP16 solution. Furthermore, the VP16 concentrations in tissues of heart, liver, spleen, lung and kidney were measured, respectively. The results were depicted in Figure 6(B–F). Intratumoral chemotherapy with the VP16 implants resulted in the low level of VP16 in tissues during the experiment. The concentrations of VP16 in liver, spleen, lung and kidney tissues were less than 800 ng/g and declined rapidly with the prolongation of time. Moreover, the VP16 concentrations decreased slowly and was maintained at low levels in heart tissue during the experiment.

## 4. Discussion

VP16 is the classical cytotoxic antineoplastic agent in the therapy of many types of cancers, usually in combination with other agents. VP16 exhibits cell cycle phase specific cytotoxicity and leads to cell cycle arrest in G2 phase and subsequent triggering of apoptosis. However, it causes dose-dependent DNA breaks and removal of VP16 can lead to fast repair of DNA breakage (Hande, 1996). The clinical use of VP16 is continuous intravenous infusion for 3 or 5 days to prolong the exposure of drug to cancer cells (Ardizzoni et al., 1999). The side effects of intravenous chemotherapy with VP16 often include bone marrow suppression, nausea, vomiting, abdominal pain, stomatitis, diarrhea, fatigue, hypotension, allergic reactions, hair loss, peripheral neuropathy and direct hepatotoxic effects (LiverTox, 2012). In addition, VP16 treatment has been associated with an increased risk of secondary leukemia (Ezoe, 2012). Thus, it is urgent to develop novel VP16-loaded delivery systems to maximize the therapeutic efficacy and minimize the systemic toxicity.

In this study, VP16 implants were prepared as cylinder by simple and reproducible direct compression method. PLLA was the main excipient in the VP16 implants. PLLA is a biocompatible, biodegradable synthetic polymer which has been safely used in clinical applications for over 30 years including dissolvable sutures, intrabone implants and soft-tissue implants (Fitzgerald et al., 2018). Furthermore, PLLA has been used as polymer matrices in the drug delivery systems (Nanaki et al., 2020; Ramesh et al., 2020). PEG4000, the other excipient in the VP16 implants was used as lubricant. Moreover, addition of hydrophilic PEG4000 can improve the dissolution and release rate of VP16 from the implants.

The HPLC method was used to determine the drug content of the VP16 implants. The result showed that the randomly tested implants (*n* = 10) had the average drug content of 36.40% which was a little less than the label claim of the drug (40%, w/w). The loss of the drug in the implants may be caused by the blending process and the acceptable measurement error. The standard deviation of drug content among the tested VP16 implants was very small. Furthermore, the SEM images of the VP16 implants demonstrated that the surface and the cross-section of the implants were almost smooth and homogenous. These results suggested that VP16 distributed uniformly in the polymer matrices.

The drug release profiles of VP16 implants were characterized by initial burst release followed by sustained-release of drug both *in vitro* and *in vivo.* Drug release kinetics determine the therapeutic effect of sustained-release drug delivery systems. The rate of drug release from polymer devices was affected by the polymer properties, enviromental conditions, drug characteristics, the shape and size of the polymer devices and drug loading (Li et al., 2010). Furthermore, it has been considered that the optimal drug release profile should be able to release a large amount of drug rapidly to reach the therapeutic concentration, and to maintain the therapeutic concentration for an extended time (Weinberg et al., 2008). In the study, we observed the initial burst release of the VP16 from the implants both *in vitro* and *in vivo*. The *in vivo* study demonstrated that the VP16 implants released approximately 20% of drug on the first day. The initial burst release of VP16 can provide a rapid ascent to the therapeutic concentration at the implantation site. Then the following sustained-release of VP16 could maintained the therapeutic concentration for a prolonged time. After being implanted into the Wistar rats intramuscularly, the duration of drug release cycle lasted up to 45 days. The prolonged exposure of VP16 at the targeted region may be able to result in the continuous inhibition of cancer cells and increase the antitumor efficacy of the VP16 implants.

DSC and FTIR analyses were carried out to test the drug–excipient compatibility of the VP16 implants. The DSC thermogram of the VP16 implants showed all endothermic events corresponding to VP16, PLLA and PEG4000. We did not observe new endothermic or exothermic peaks in the DSC curve of VP16 implants, indicating that there was no chemical interaction between VP16 and the excipients. The FTIR study determine the drug–excipient interaction at the level of functional groups (Gao et al., 2019). From the FTIR spectra of VP16 implants, we detected the typical infrared absorption bands that also be visualized in pure powders of VP16, PLLA and PEG4000, and the physical mixture of VP16 and excipients. Moreover, we did not find new bands in FTIR spectra of the VP16 implants. Therefore, the results of DSC and FTIR analyses supported that no chemical interactions occurred among VP16, PLLA and PEG4000 in the implants. The VP16 implants exhibited good compatibility between the drug and the excipients.

Several studies have revealed that direct injection of chemotherapeutics into tumor tissue resulted in high tumor drug concentrations (Lu et al., 2015). However, the leakage or backflow of drug was observed when intratumoral chemotherapy with the diluted drug solution (Hohenforst-Schmidt et al., 2013). In this study, the mice bearing Lewis lung carcinoma received intratumoral chemotherapy with the solid sustained-release VP16 implants at the dose of 75 mg/kg. The implants did not leak from the tumor during the whole experiment.

We used the accurate and precise UPLC-MS/MS method to detect the concentrations of VP16 in plasma and tissues. The plasma drug levels of VP16 were below 500 ng/mL during the experiment after intratumoral chemotherapy with the VP16 implants. We think the plasma VP16 level is safe for the body because the clinical practices have revealed that plasma VP16 concentrations of 0.7–2.0 µg/mL were associated with cytotoxicity and higher plasma concentrations lead to additional myelosuppression (Hande, 1996).

We observed high drug concentration in tumors on the first day after intratumoral chemotherapy with the VP16 implants. This was related to the initial burst release of VP16 from the implants. Then the high level of VP16 in tumor maintained for several days and declined slowly with the extension of time. The control mice were administered single intraperitoneal injection of VP16 solution. The result indicated that only a small fraction of VP16 accessed the tumor site. The low drug concentration in tumor could not achieve the antitumor effect. In clinical practice, continuous infusion of VP16 may increase the drug concentration in tumor, but the higher systemic toxicity is unavoidable (Ardizzoni et al., 1999).

We then tested the distribution of VP16 in tissues of heart, liver, spleen, lung and kidney, the results suggested that intratumoral chemotherapy with the VP16 implants resulted in accumulation of small amounts of VP16 in tissues. In our previous research, the nude mice bearing A549 lung cancer cells were intratumorally inserted VP16 implants at the dose of 75 mg/kg. During the observation period, the body weights of the mice increased gradually and had no significant difference compared with the mice without treatment (Gao et al., 2017). On the basis of the previous data, we consider that distribution of VP16 in the organ tissues after intratumoral chemotherapy with the VP16 implants have no toxic effect on the body. We are going to carry out the further research to support this point of view.

Taken together, the VP16 implants showed the following advantages: (і) the method of preparation of VP16 implants was simple and reproducible. (іі) the solid VP16 implants were easy to preserve. (ііі) the VP16 implants demonstrated good compatibility of the drug and the excipients. (іv) the drug release profiles may maximize the anticancer efficacy of the implants. (v) local chemotherapy with the VP16 implants yielded high drug centrations at the target site and avoided excessive drug accumulation in normal tissues.

## 5. Conclusion

In the present study, we prepared PLLA based VP16 implants with drug content of 36.40%. Both *in vitro* and *in vivo* drug release profiles of the implants were characterized by high initial burst release followed by sustained-release of the VP16. The DSC and FTIR analyses indicated good compatibility of VP16 and the excipients. Intratumoral chemotherapy with the VP16 implants resulted in high drug concentrations in tumor tissues for a prolonged time. Moreover, the low VP16 concentrations were detected in the plasma and normal organ tissues and the VP16 level declined obviously with the extension of time.

## Supplementary Material

Supplemental Material
