# Supplementary figures and images for: Biodistribution of etoposide via intratumoral chemotherapy with etoposide-loaded implants
Source: Drug Deliv. 2020 Jul 2;27(1):974–82. doi: 10.1080/10717544.2020.1787558 (PMC8216434; doi:10.1080/10717544.2020.1787558)

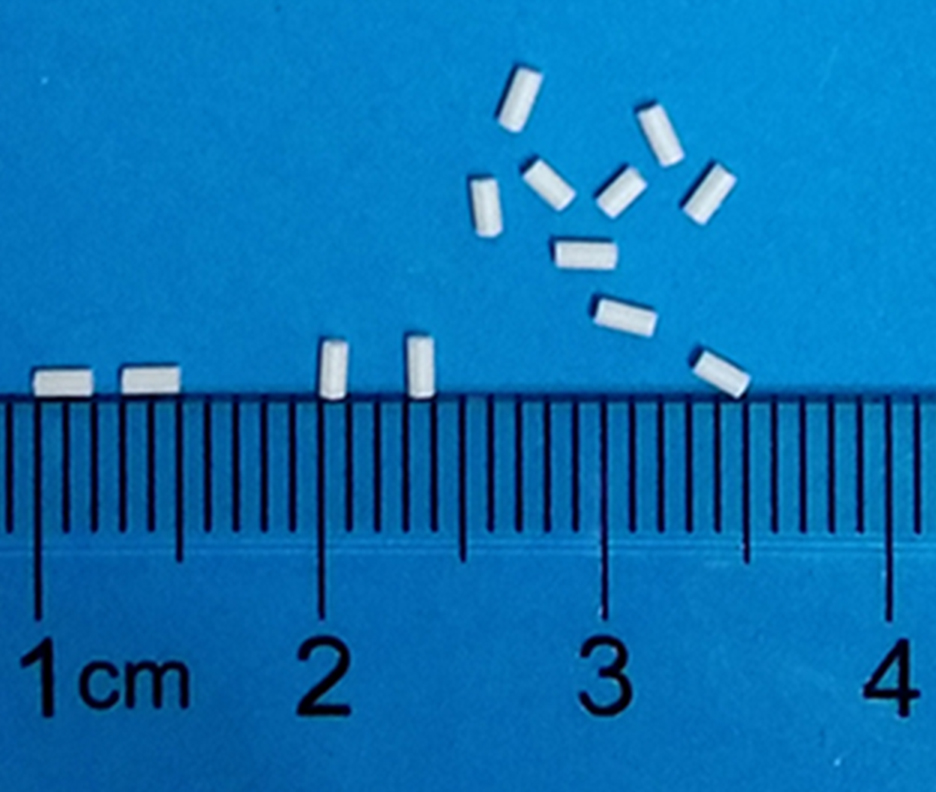

Supplement: Supplemental Material [file IDRD_A_1787558_SM5165.zip › Figure S1.jpg]

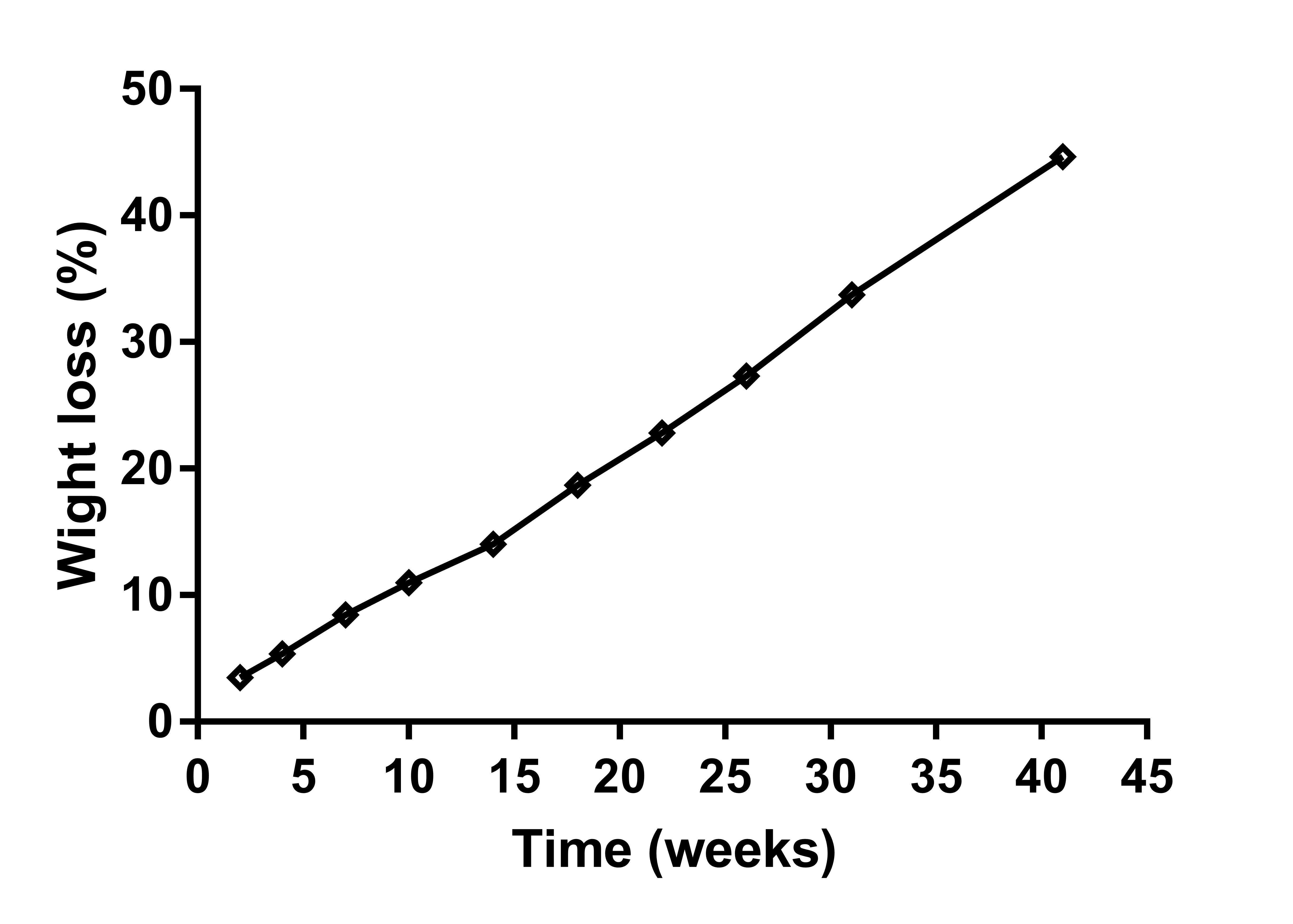

Supplement: Supplemental Material [file IDRD_A_1787558_SM5165.zip › Figure S2.jpg]
